# Testes and duct deferens of mice during space flight: cytoskeleton structure, sperm-specific proteins and epigenetic events

**DOI:** 10.1038/s41598-019-46324-3

**Published:** 2019-07-05

**Authors:** Irina V. Ogneva, Maria A. Usik, Sergey S. Loktev, Yuliya S. Zhdankina, Nikolay S. Biryukov, Oleg I. Orlov, Vladimir N. Sychev

**Affiliations:** 1 Cell Biophysics Laboratory, State Scientific Center of Russian Federation Institute of Biomedical Problems of the Russian Academy of Sciences, Khoroshevskoyoe shosse, 76a, Moscow, 123007 Russia; 2 I.M. Sechenov First Moscow State Medical University, 8-2 Trubetskaya St., Moscow, 119991 Russia

**Keywords:** Cytoskeleton, Epigenetic memory

## Abstract

To analyze the effect of gravity on the structure of germinal tissues, we examined tissues of the testes and duct deferens of mice that were exposed to space flight conditions for 21–24 days (experiment Rodent Research-4, SpaceX-10 mission, February 2017, USA). We evaluated the levels of cytoskeletal proteins, sperm-specific proteins, and epigenetic events; in particular, we evaluated levels of 5-hydroxymethylcytosine and of enzymes that regulate DNA methylation/demethylation. We did not detect changes in the levels of cytoskeletal proteins, sperm-specific proteins, DNA-methylases, DNA demethylases, DNA acetylases, or histone deacetylases. However, there were changes at the gene expression level. In particular, there was an increase in the demethylase *Tet2* and a decrease in the histone deacetylase *Hdac1*. These gene expression changes may be of key importance during the early period of readaptation since they could lead to an increase in the expression of target genes.

## Introduction

The role of gravity in the early development of mammals, particularly during prenatal development, remains unclear despite a number of previous studies^1–8^.

It is known that the exposure of female rats to zero gravity in the second half of pregnancy does not cause significant changes in embryos^3,6^, which may be due to the damping of the external mechanical field by the amniotic fluid. On the other hand, the preimplantation development and early stages of gastrulation may be more sensitive to changes in external mechanical stress. For example, pregnancy was not achieved in mice during space flight conditions, which researchers attributed to the lack of implantation and, as a result, the abortion of the preimplantation embryos^7^.

In addition, there is every reason to believe that germ cells can change their structure in microgravity conditions. For male germ cells, it is known that the speed of the movement of the mouse spermatozoa decreases after a 7-day antiorthostatic hanging, which simulates the effects of weightlessness^9^; although, for the sperm of sea urchin in space flight (STS-81, STS-84), FP130 protein phosphorylation, which is associated with the activation of motility, occurred 3–4 times faster than in conditions of 1 g^10^. Additionally, the expression of genes encoding pro-apoptotic proteins increased^11^, the differentiation potential of progenitor cells changed^12^, and the efficiency of spermatogenesis decreased^13,14^. However, the causes of such changes in sperm are not at all clear.

Our previous data suggested that after a 30-day antiorthostatic suspension, changes in the patterns of cytoskeletal proteins in mouse spermatozoa and testes were not observed, although the relative mRNA levels of the corresponding genes changed^15^. It is known that the external mechanical field plays an important role in establishing the patterns of expression of various genes, primarily those encoding cytoskeletal proteins and the associated signaling cascades, in different cell types, including stem cells^16–22^. However, how an external signal that the mechanical field has changed is transduced and leads to a change in gene expression remains completely unclear.

In higher mammals, there are a large number of methods for regulating gene expression, such as various posttranslational modifications of histones, chromatin remodeling, RNA interference, and DNA methylation, but most of these processes have hardly been studied under microgravity conditions. Few and partially contradictory data are associated with the determination of the methylation levels in different cell types in conditions of microgravity: hypermethylation took place in rice germs under space flight conditions^23^, hypomethylation was observed in human T lymphocytes^24^, and in lymphoblastoid cells, there was a change in the methylation level and in the hydroxymethylation level^25^. The data from our previous study showed that in space flight conditions, the total level of DNA methylation in the heart and lung tissues of mice increased by 21% and 32%, respectively, which correlated with changes in gene expression levels^26^.

Therefore, the purpose of our work was to evaluate the levels of the cytoskeletal proteins and the mRNA levels of the genes that encode them in the testes and duct deferens of mice whose tissues were fixed in space flight conditions. In addition, we sought to evaluate epigenetic events, in particular, the levels of 5-hydroxymethylcytosine and of the enzymes that regulate DNA methylation/demethylation.

## Results

### The levels of the cytoskeletal proteins and the corresponding mRNA

**Figure 1:**
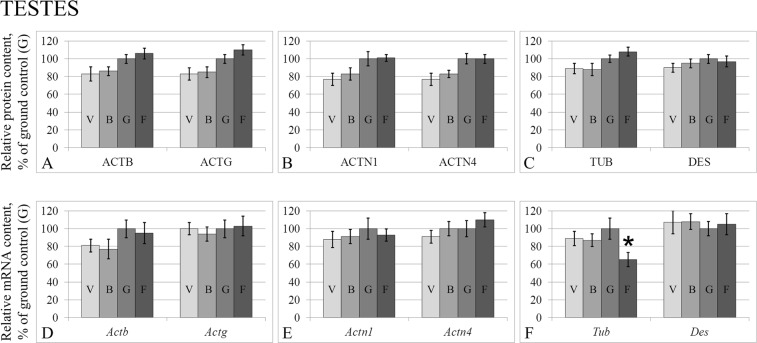
Relative protein contents of cytoskeletal proteins in the testis tissue (**A–C**) and according mRNA levels (**D–F**). ACTB – Beta-actin. ACTG – Gamma-actin. ACTN1 – Alpha-actinin-1. ACTN4 – Alpha-actinin-4. TUB – Beta-tubulin. DES – desmin. V – vivarium control, B – basal control, G – ground control, F – flight group. *p < 0.05 in comparison with the G group.

**Figure 2:**
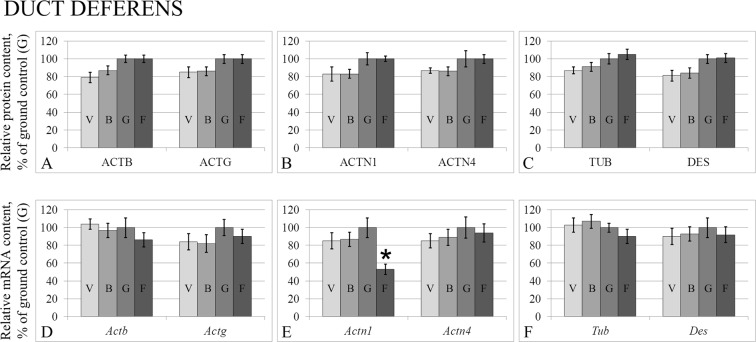
Relative protein contents of cytoskeletal proteins in the duct deferens tissue (**A**–**C**) and according mRNA levels (**D**–**F**). ACTB – Beta-actin. ACTG – Gamma-actin. ACTN1 – Alpha-actinin-1. ACTN4 – Alpha-actinin-4. TUB – Beta-tubulin. DES – desmin. V – vivarium control, B – basal control, G – ground control, F – flight group. *p < 0.05 in comparison with the G group.

In the testes (Fig. 1) and the duct deferens (Fig. 2), no changes in the relative levels of beta-actin, gamma-actin, alpha-actinin-1, alpha-actinin-4, beta-tubulin and desmin were observed. There were also no changes in the relative levels of the corresponding mRNA, except for tubulin in the testes (Fig. 1F), where the mRNA content of the flight group was lower than that of the control group by 35% (p < 0.05) and alpha-actinin-1 in the duct deferens (Fig. 2E) in group F, where the *Actn1* mRNA level was 47% lower than that of the control group (p < 0.05).

### Levels of proteins that are specific to different stages of spermatogenesis and their corresponding mRNA levels

**Figure 3:**
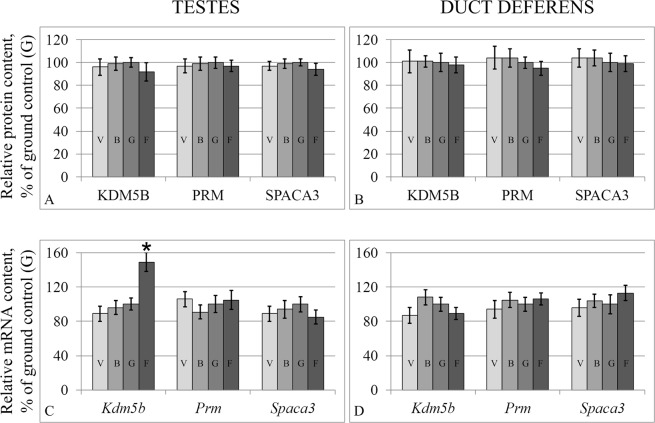
Relative protein contents of sperm-specific proteins in the testis (**A**) and duct deferens tissues (**B**) and according mRNA levels (**C** – testes, **D** – duct deferens). KDM5B (Jarid1B) – specific for spermatogonia; PRM (protamine) – specific for spermatids; Spaca 3 – specific for mature sperm. V – vivarium control, B – basal control, G – ground control, F – flight group. *p < 0.05 in comparison with the G group.

Similar to cytoskeletal proteins, the levels of proteins that are specific to early spermatogonia (KDM5B), spermatids (PRM1) and mature spermatozoa (SPACA3) did not differ in any of the study groups (Fig. 3), both in the testes and in the duct deferens. The mRNA levels were also the similar, with the exception of the *Kdm5B* mRNA level, which in the flight group in the testes (Fig. 3C) was higher than that of the control by 49% (p < 0.05).

### The relative levels of 5-hydroxymethylcytosine

**Figure 4:**
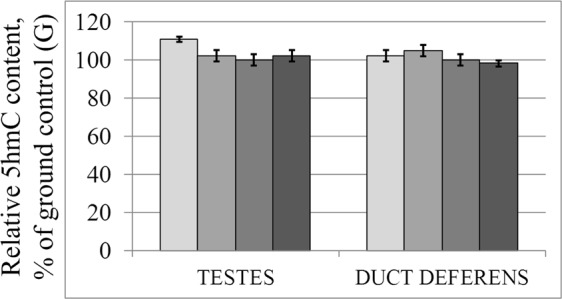
Relative 5-hydroxymethycytosine level in the testis and duct deferens tissues. V – vivarium control, B – basal control, G – ground control, F – flight group.

The levels of 5-hydroxymethylcytosine did not differ in the study groups, both in the testes and in the duct deferens (Fig. 4).

### Methylases/demethylases and acetylases/histone deacetylases and their mRNA levels

**Figure 5:**
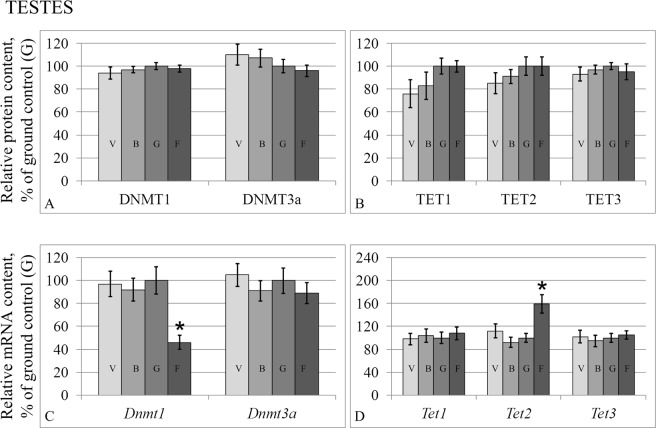
Relative protein contents of methylation/demethylation fermentas in the testis tissue (**A,B**) and according mRNA levels (**C,D**). DNMT1 – S-phase methylase DNMT3a – *de novo* methylase. TET 1,2,3 – demethylases of the TET-family. V – vivarium control, B – basal control, G – ground control, F – flight group. *p < 0.05 in comparison with the G group.

**Figure 6:**
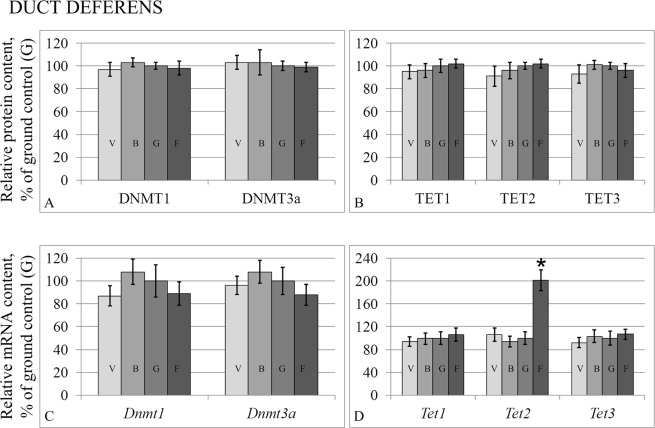
Relative protein contents of methylation/demethylation fermentas in the duct deferens tissue (**A,B**) and according mRNA levels (**C,D**). DNMT1 – S-phase methylase DNMT3a – *de novo* methylase. TET 1,2,3 – demethylases of the TET-family. V – vivarium control, B – basal control, G – ground control, F – flight group. *p < 0.05 in comparison with the G group.

The levels of the S-phase methylase DNMT1 did not change in the testes (Fig. 5A) or in the duct deferens (Fig. 6A); however, the expression level of the corresponding gene in the testes (Fig. 5C) of group F was lower than that of group G by 54% (p < 0.05). At the same time, there was no change in the protein or mRNA levels of the *de novo* methylase DNMT3a either in the testes or in the duct deferens.

At the same time, the relative protein and mRNA levels of the TET family demethylases of group F in the testes (Fig. 5B) and duct deferens (Fig. 6B) did not change during flight, except for TET2 mRNA. In the flight group, the relative TET2 mRNA level increased in the testes (Fig. 5D) by 59% (p < 0.05) and in the duct deferens (Fig. 6D) by 101% (p < 0.05).

**Figure 7:**
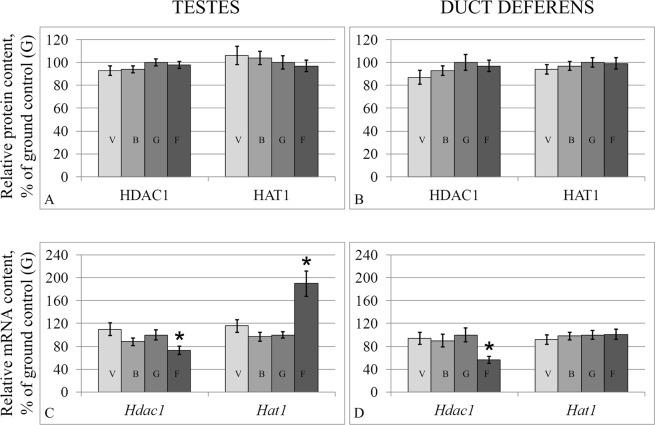
Relative protein contents of acetylation/deacetylation fermentas in the testis (**A**) and duct deferens tissues (**B**) and according mRNA levels (**C**,**D**). HAT1 – histone acetylase. HDAC1 – histone deacetylase V – vivarium control, B – basal control, G – ground control, F – flight group. *p < 0.05 in comparison with the G group.

After flight, the level of HDAC1 in the testes (Fig. 7A) and the duct deferens did not change (Fig. 7B), although the relative level of the mRNA decreased by 27% (p < 0.05) (Fig. 7C) and 44% (p < 0.05) (Fig. 7D), respectively.

The level of the histone acetylase HAT1 did not change in the study groups (Fig. 7A,B); however, the relative level of the corresponding mRNA increased after flight in the testes (Fig. 7C) by 90% (p < 0.05), although in the duct deferens it remained unchanged.

## Discussion

Studies of the influence of weightlessness on the structure and function of male germ cells not only are applied research but also are of fundamental importance to the understanding of the evolutionary aspects of ontogenesis. However, on Earth, all such studies have limitations that are inherent to all modeling experiments. Therefore, the data from tissue studies that were recorded directly in space flight conditions are of particular interest.

In this study, we had the opportunity to study the duct deferens and the testes of mice that were in zero gravity for approximately 23 days and were euthanized there.

The results suggested that there was no change in the levels of the studied cytoskeletal proteins (beta- and gamma-actin, alpha-actinin 1 and 4, beta-tubulin and desmin) in the flight group, although there was a decrease in *Actn1* mRNA in the duct deferens and in beta-tubulin in the testes. Interestingly, in the heart and lungs after a 34–37-day flight (in the Rodent Research-1 experiment that also performed tissue fixation under weightlessness), there were practically no changes in the levels of cytoskeletal proteins, although there were significantly more changes in the mRNA levels^26^. The latter may be because in the early stages of space flight, the heart and lungs are subjected to an increase of external mechanical stress due to a volume shift in the cranial direction, unlike the testes and the duct deferens. However, it should be noted that testes tissues have high levels of cellular heterogeneity, which does not allow for the identification of the contributions of individual cell types to the results that we obtained. Therefore, we decided to examine the relative content of proteins and mRNA which expression correlates with different degrees of sperm maturity, both in the duct deferens and in the testes.

KDM5B (a histone demethylase that demethylates ‘Lys-4’ of histone H3) was chosen as a marker for early spermatogonia, since their differentiation into spermatocytes requires inactivation of this gene^27^. In the duct deferens, we did not observe changes in the protein levels and mRNA levels of the encoding gene, *Kdm5B*. However, in the testes, despite the absence of a change in the protein level, the mRNA level of this gene increased in the flight group, which may indicate a decrease in the differentiation potential and, accordingly, in the mature forms of sperm. We observed a similar effect after a 30-day antiorthostatic suspension, where a decrease in the number of the mature forms of sperm correlated with an increase in the mRNA level of the *Kdm5B* gene^15^. However, in this case, we did not note changes in the levels of protamines, the expression of which is highest in spermatids^28^, as well as sperm lysozyme-like protein 1 (SLLP1, Spaca3), which is localized to the acrosome of mature spermatozoa^29^. Perhaps this is because more mature forms of sperm are localized in the caudal epididymis, which was not available for analysis.

Thus, despite the absence of changes in the levels of cytoskeletal and sperm-specific proteins, changes in the expression of some proteins were observed.

On the one hand, this situation correlates with our previous data after Rodent Research-1 mission^26^ and may indicate a change in the efficiency of translation and/or proteolysis. Earlier, we proposed the role of actin-binding proteins in the cell mechanosensitivity at early stages of microgravity^30^. One of them, alpha-actinin-1 interacts with phospholipase D in the cortical cytoskeleton, and it may regulate efficiency of translation^31^.

On the other hand, the question remains: what caused the changes in the observed gene expression? The reasons for the changes in expression could be associated with a wide range of factors, such as histone modifications, but for higher mammals, it is most likely because of a change in the DNA methylation levels of CpG-islands in the promoter regions of genes.

In this study, we attempted to analyze the total DNA methylation level by restriction analysis and, accordingly, CG-islands in the promoter regions of the cytoskeletal genes were studied; however, the isolated genomic DNA was not of a high enough quality to conduct such an analysis.

Nevertheless, we were able to evaluate the level of 5-hydroxymethylcytosine (5hmC), which is an intermediate product between the fully methylated and fully demethylated states^32^. There were no changes in the 5-hydroxymethylcytosine levels in the testes or duct deferens. It should be noted that in the ovaries of mice after 23 days of antiorthostatic suspension, there were also no changes in the 5hmC levels, although we noted a change in the expression of the gene encoding one of the TET-family demethylases, TET2. Therefore, in this work, we also decided to examine the content and expression of genes encoding key methylases/demethylases.

The protein content of the S-phase methylase DNMT1, *de novo* methylase DNMT3a, and demethylases of the TET family (TET1, TET2, and TET3) of the flight group both in the testes and duct deferens remained at the same level as the control group. However, the gene expression changed in some cases. For example, the expression of the *Dnmt1* gene decreased in the testes, and the expression of *Tet2* increased both in the testes and in the duct deferens, and in the previous experiment that examined the ovaries of mice after antiorthostatic suspension^33^. Since the DNMT1 methylase and proteins of the TET family are part of a complex that interacts with DNA^34^, it can be assumed that a decrease in the expression of one of the participants of the complex could lead to a decrease in the number of active complexes and, as a consequence, to a decrease in the DNA demethylation level. On the other hand, the acetylation of TET leads to an increase in its demethylation activity, and the deacetylation of HDAC1/2 proteins leads to the inhibition of their activity involving demethylases^34^. Interestingly, as previously observed^33^, an increase in *Tet2* expression in the flight group correlated with a decrease in *Hdac1* deacetylase expression. At the same time, a decrease in *Dnmt1* methylase expression was accompanied by an increase in *Hat1* histone acetylase expression in the testes of mice of the flight group.

In summary, the expression of the *Hdac1* deacetylase gene was reduced in the duct deferens, while the mRNA levels of the acetylase *Hat1* remained constant; and in the testes, while the expression of the deacetylase was reduced, the expression of the acetylase was increased. Histone acetylation can regulate the efficiency of transcriptional activation both by directly changing the charge^35^ and causing chromatin remodeling and by the recruitment of transcriptional activators^36–38^. Therefore, in a case where the expression level of the acetylases/deacetylases changed at the translational level, we could expect to see a greater change in the expression of other genes, such as those encoding cytoskeletal proteins.

## Conclusions

In general, in this study of the duct deferens and testes tissues of mice that were euthanized under weightless conditions, we did not detect changes in the levels of cytoskeletal proteins, sperm-specific proteins, DNA methylases, DNA demethylases, DNA acetylases and histone deacetylases. This may indicate that during 3 weeks of space flight, an adaptive protein profile formed in the germinative tissues of male mice.

However, we did observe changes in the gene expression, especially of *Tet2* demethylase (an increase) and of the histone deacetylase *Hdac1* (a decrease). An increase of mechanical stress, such as the increase that is associated with a return to Earth’s gravity conditions, can trigger the realization of this pattern at the protein level and subsequent changes of cells structure and functions. That is why further detailed research is required to develop effective strategies to protect human health during the early period of readaptation to varying gravity after spending a long period under weightless conditions.

## Materials and Methods

### Experimental design

In the “Rodent Research-4” (RR-4) experiment, C57BL/6J mice obtained from the Jackson Laboratory (Jackson Laboratory, Bar Harbor, ME) that were 9–12 weeks old at the time of launch were used. The animals were placed in a transport container on February 18, 2017. For four days, the transport container remained in the capsule of the Dragon SV on the launch pad. The SpaceX-10 spacecraft was launched on February 23, 2017. The animals were transferred from the transport container into an animal habitat unit onboard the ISS on February 25, 2017. During flight conditions, the animals received food and water *ad libitum*. All description of transport container and habitat with animal welfare were provided by Ronca A.E. *et al*.^39^.

The animals in the flight group (F) were kept in microgravity for 21–24 days, after which they were euthanized. Euthanasia was made by cardiac puncture and blood collection with pre-made anesthesia. Then, the carcasses were wrapped in two layers of foil and placed in plastic bags (so that each carcass was completely surrounded by ice), which were inserted into piles for storage at low temperature. The period between euthanasia and freezing of the carcass was approximately 2 minutes. The carcasses were stored in the MELFI freezer until returning to Earth.

The animals in the basal control group (B) were euthanized in normal laboratory conditions shortly after the launch of SpaceX-10. Animals of the vivarium control group (V) were kept in standard vivarium conditions and received water and food *ad libitum*. The animals in the ground control group (G) were euthanized in the laboratory after staying in microgravity conditions while being recorded with a camera (at the John F. Kennedy Space Center, USA), simulating the same environmental conditions as on the ISS.

The schedule for working with the animals of groups B, V and G corresponded to the schedule for working with group F.

All animal procedures were approved by the Ames Institutional Animal Care and Use Committee (Ames, USA).

Organs were isolated from the frozen carcasses on Earth. The right testis (number of animals: B - 10, V - 10, G - 10, F - 6) and duct deferens (number of animals in the groups: B - 10, V - 10, G - 10, F - 6) were frozen in liquid nitrogen for determination of protein content; the left testis (number of animals: B - 10, V - 10, G - 10, F - 8) and duct deferens (number of animals: B - 10, V - 10, G - 10, F - 8) were placed in the RNAlater Stabilization Solution (Qiagen, Germany) for future work with nucleic acids. For each group of animals, we examined the biomaterial of a minimum of 6 animals.

The samples, in accordance with the NASA-Roskosmos protocol “Utilization Sharing Plan on-board ISS” (signed on July 18, 2013), were delivered to Russia on dry ice without defrosting, which was ensured by the use of temperature sensors.

### Determination of protein content by western blot

Isolation of total protein from the frozen tissues and denaturing electrophoresis were performed according to the Laemmle method using a Bio-Rad system (USA). The protein concentration of each sample was measured, and the same amount of protein was loaded into each well. Transfer to the nitrocellulose membrane was performed according to Towbin *et al*.^40^.

To identify each protein, specific antibodies were used at the dilution recommended by the respective manufacturer. Specific antibodies raised in mice were used to examine the cytoskeletal proteins: beta-actin (at a dilution of 1: 500), gamma-actin (1: 200), alpha-actinin-1 (1: 200), alpha-actinin-4 (1: 200), beta-tubulin (1: 200), and desmin (1: 200) (all from Santa Cruz Biotechnology, Inc., USA). Specific antibodies raised in rabbits for the listed proteins were used at the following dilutions: S-phase methylase DNMT1 (1: 1000), *de novo* methylase DNMT3a (1: 2000), 5-methylcytosine hydroxylase (demethylase) TET1 (2 µg/ml), TET2 (1: 1000), TET3 (1: 1000), histone acetylase KAT1/HAT1 (1: 1000), and histone deacetylase HDAC1 (1: 1000) (Abcam, UK); JARID1B (KDM5B) (1: 100) (Bioss Antibodies, USA); protamine (1: 250) (Sigma, Germany); and SPACA3 (1: 1000) (OriGene, USA). Biotinylated goat antibodies against mouse IgG (1:20,000) (Sigma, Germany) and rabbit IgG (1:10,000) (Jackson ImmunoResearch Lab, Inc., USA) were used as secondary antibodies. Further, all membranes were treated with a solution of streptavidin conjugated with horseradish peroxidase (Sigma, Germany) at a dilution of 1:10,000. Protein bands were detected using 3,3′-diaminobenzidine (Merck, USA), and the data were analyzed using ImageJ.

### Determination of the mRNA relative content by quantitative PCR

Total RNA from tissues, stained in the RNAlater Stabilization Solution, was isolated using the RNeasy Micro Kit (Qiagen, Germany) according to the manufacturer’s instructions. Reverse transcription was performed using d (T)_15_ as a primer and 500 ng of RNA. The relative mRNA content of the studied genes was estimated using real-time PCR with primers selected by the Primer3Plus program (Table 1), the results were processed using the 2(−Delta Delta CT) method^41^.

**Table 1:** Primer sequences and product sizes.

Gene	Primer sequence, forward/reverse (5′…3′)	Product size, bp
*Actb* (beta-actin)	*gctgcgttttacaccctttc*/*gtttgctccaaccaactgct*	218
*Actg* (gamma-actin)	*ctggtggatctctgtgagca*/*tcaggagggaagaaaccaga*	184
*Actn1* (alpha-actinin 1)	*ggtcagcagcaacctcctc*/*tctttctccaccttctctcca*	167
*Actn4* (alpha-actinin 4)	*accctgaacagactcccttg*/*gatcgacaagcctccatctc*	168
*Tubb2b* (beta-tubulin 2B)	*ggcagcaagaagctaacgag*/*cgaacacgaagttgtctggc*	302
*Des* (desmin)	*gtgaagatggccttggatgt*/*cgggtctcaatggtcttgat*	182
*Kdm5b*(lysine (K)-specific demethylase 5B, JARID1B)	*agtggctttcctgttcgaga*/*aagcacatgcccacatacaa*	173
*Prm1*(protamine 1)	*atggccagataccgatgct*/*cgagatgctcttgaagtctgg*	231
*Spaca3*(sperm acrosome associated 3, SLLP1)	*caaggccaaggtcttcagtc*/*tcagcttcatgatccacagc*	150
*Dnmt1* (S-phase methylation)	*ccggaaactcacttggacga*/*tttggcagctggatctctgg*	90
*Dnmt3A* (*de novo* methylation)	*agagcgctttgactccacat*/*ggaccaggaaaaacaaacga*	150
*Tet1* (cytosine demethylase)	*gtgtgggtcgatggctctat*/*cttattcccaccaccgctaa*	208
*Tet2* (cytosine demethylase)	*gttctcaacgagcaggaagg*/*tgagatgcggtactctgcac*	185
*Tet3*(cytosine demethylase)	*ttctatccgggaactcatgg*/*ccaggccaggatcaagataa*	226
*Hat1* (histone aminotransferase 1)	*agagtgccgtggagaagaaa*/*tttcatcatccccaaagagc*	150
*Hdac1* (histone deacetylase 1,2,3,4,6,9)	*ccatgaagcctcaccgaat*/*caaacaccggacagtcctca*	226

### Determination of the 5-hydroxymethylcytosine (5hmC) content in DNA by the dot blot method

To determine the methylation levels, total DNA was isolated from the tissues, stained in the RNAlater Stabilization Solution, using a DNA extraction kit (Sintol, Russia) based on the phenol/chloroform method according to the manufacturer’s instructions. The isolated DNA was applied to a nitrocellulose membrane, for the preliminarily measurement of the concentration and for denaturing (+95 °С for 5 min and then +4 °С for 3 min), at three dilutions −1 μg, 500 ng, and 200 ng. The membranes were air-dried, welded to the membrane using ultraviolet light and incubated in 4% skim milk overnight at +4 °C.

To evaluate the 5hmC content, specific antibodies raised in rabbits (Abcam, United Kingdom) were used at a dilution of 0.5 μg/ml in accordance with the manufacturer’s instructions. Biotinylated goat anti-rabbit IgG antibodies (Jackson ImmunoResearch Lab., Inc., USA) were used as secondary antibodies at a dilution of 1:10,000. Then, the membranes were treated with a streptavidin solution that was conjugated with horseradish peroxidase (Sigma, Germany) at a dilution of 1:10,000. Dots were detected using 3,3′-diaminobenzidine (Merck, USA). ImageJ was used to analyze the data.

### Statistical analysis

The results obtained were analyzed by ANOVA, using a post hoc t-test with significance level p < 0.05 to assess the significance of the differences between the groups. The data are presented as M ± SE, where M is the arithmetic average and SE is the error of the mean.

All methods were performed in accordance with the relevant guidelines and regulations.

## Data Availability

All data generated or analyzed during this study are included in this article.
